# The assessment of chemical composition and biological activity of faba bean pods as a potential feed additive utilized in piglets nutrition

**DOI:** 10.1038/s41598-024-84925-9

**Published:** 2025-01-07

**Authors:** Inna Vlasova, Philip Krüsselmann, Yuliia Kostenko, Maciej Obrębski, Sebastian Granica, Wilfried Vahjen, Jürgen Zentek, Marcin Równicki, Jakub P. Piwowarski

**Affiliations:** 1 Microbiota Lab, Department of Pharmaceutical Biology, Medical University of Warsaw, 1 Banacha St., Warsaw, 02-097 Poland; 2 Institute of Animal Nutrition, Department of Veterinary Medicine, Freie Universität Berlin, Berlin, Germany

**Keywords:** Faba bean pods, Food by-product, Polyphenols, Digestive enzymes, Antimicrobials, Drug discovery, Sustainability

## Abstract

The study addresses the utilization of food waste by-products from faba bean (*Vicia faba* L.) pods (FBP) as an alternative feed supplement to promote sustainable piglet growth by reducing antimicrobial use. Objectives include evaluation of FBP in terms of nutritional components (proximate composition, fibres, minerals), phytochemical composition (total phenols, HPLC-MS profiling), and in vitro biological activities. Air-dried FBP from the cultivar ‘Bizon’ contained high levels of crude protein (144 g/kg), dietary fibre (413 g/kg), potassium (27.8 g/kg), and iron (126 mg/kg). Phytochemical analysis of methanolic extract from FBP revealed significant levels of polyphenols, including vestitol, piscidic acid, hydroxyeucomic acid, quercetin, and kaempferol glycosides with no detectable tannins. The extract showed negligible activity against porcine digestive enzymes (α-amylase, lipase, and trypsin) (IC_50_ > 4 mg/mL) and demonstrated a dose-dependent antibacterial activity against *Escherichia coli* and *Salmonella enterica* in concentrations of 1–8 mg/mL. The extract had low cytotoxicity (IC_50_ = 432.6 µg/mL) against IPEC-J2 – cells derived from porcine jejunal epithelium. The results indicate that FBP ‘Bizon’ is a valuable source of bioactive compounds with antibacterial properties, without adverse effects on porcine enzymes or IPEC-J2 cells, supporting its potential as a sustainable feed in piglet nutrition, in line with circular economy concepts.

**Supplementary Information:**

The online version contains supplementary material available at 10.1038/s41598-024-84925-9.

## Introduction

Global food waste is a cross-cutting issue that extends from agricultural production to the landfill, generating a loss of up to 40% of the initial product^1^. On the other hand, agro-industrial by-products and food production residues are interesting, renewable sources of potential bioactive natural products. A significant portion of by-products consists of plant-based materials, given that only a limited segment of the entire plant is edible.

One example of a plant that generates large amounts of by-products is faba bean (*Vicia faba* L.)^2^. Faba bean is a versatile legume cultivated all over the world and is considered as a good source of proteins, dietary fibres, and trace elements^3,4^. Besides, the plant has been found to be rich in bioactive compounds, such as phenolics, which are known for their antioxidant, anti-inflammatory, and antimicrobial properties^5^. However, the level of incorporation may vary based on the presence of antinutrients^4^. Faba bean is grown primarily for its edible seeds^6^; however, the plant has many other parts potentially containing bioactive compounds. Throughout the process, from harvesting to consumption, various plant residues such as stems, leaves, seed husks, and pods are generated and remain as by-products. This implies that up to 60% (w/w) of the aboveground biomass remains unused. In this context, the empty faba bean pods (FBP) constitute about 20% (w/w) of the total harvest residues^7^. Given the functional and economic importance, the global production of faba bean is most likely to increase in the future^2^. This means that the amount of by-products that are not utilized in any way will also increase. FBP are mainly composed of dietary fibres but also contain high amounts of bioactive phytochemicals, offering many benefits to enhance the profitability of this underutilized by-product, for example, as feed additives in animal nutrition. The fibre content is of particular interest in swine nutrition, as the ability to digest it can influence gut health and nutrient absorption. In piglets, the fibre is less digestible due to their immature digestive systems and lower enzymatic activity, which limits the breakdown of fibrous materials^8^. High levels of dietary fibre can reduce the overall digestibility of nutrients like protein and fat, potentially leading to slower growth rates^9^. However, the inclusion of moderate levels of faba bean pod fibre has been shown to support the development of beneficial gut microbiota and improve intestinal health^10^. In older pigs, however, the digestibility of fibre improves due to a more developed digestive system and increased microbial fermentation capacity in the large intestine^11^. Therefore, it is important to revalorize FBP as valuable feed components in piglet nutrition, to fully exploit the potential of this biomass and reduce food waste. One of the most important diseases among farm animals is postweaning diarrhoea, where new preventive and therapeutic strategies are urgently needed^12^. The aetiology of postweaning diarrhoea is multifactorial, although it is often associated with infections caused by Gram-negative bacteria: *Escherichia coli* and *Salmonella enterica*^13,14^. Initially, antibiotic growth promoters were developed for healthy animals, with the prevention of diarrhoea being a beneficial side effect; however, due to their risk for spreading antibiotic resistance, the European Union introduced a ban on these substances in 2006 (Regulation 1831/2003/EC). As a result, numerous concepts have been developed that include the feed composition, its components and the area of feed additives. However, it is still necessary to investigate new substances that could potentially lead to a reduction in diarrheal diseases in piglets^15^.

The present study comprehensively investigated the phytochemical and nutritional content of FBP. Considering the attributed anti-nutritional properties of faba bean and the potential application of its pods as functional feed compounds, the in vitro biological activity was evaluated, including the influence of FBP on porcine digestive enzymes and antimicrobial activity against porcine gastrointestinal pathogens.

## Results

### Nutritional composition

The nutritional analyses were conducted using methods recommended by the Association of German Agricultural Inspection and Research Institutes with air-dried FBP. As presented in Table 1, the dry matter content was found to be 911 g/kg air-dried FBP. The average values of crude ash, fat, protein, and fibre were 66.3, 7.04, 144, and 207 g/kg (air-dry weight), respectively. The plant material included 243 g/kg acid detergent fibre and 326 g/kg neutral detergent fibre (Table 1). The total amount of dietary fibre was 413 g/kg, consisting of 328 g/kg of insoluble dietary fibre and 85 g/kg of soluble dietary fibre (Table 1). Additionally, the FBP samples contained several important minerals, including potassium, phosphorus, calcium, sodium, magnesium, and some essential trace elements such as iron, copper, zinc, and manganese (Table 1).

**Table 1:** Proximate composition, total fibre, and mineral contents in air-dried faba bean pods.

Proximate composition	g/kg
Dry matter	911
Crude ash	66.3
Crude fat	7.04
Crude protein	144

Fibre	g/kg
Crude fibre	207
Acid detergent fibre	243
Neutral detergent fibre	326
Dietary fibre (DF)	413
Insoluble DF	328
Soluble DF	85

Minerals	g/kg
Potassium	27.8
Phosphorus	2.75
Calcium	2.42
Sodium	0.53
Magnesium	1.71

	mg/kg
Iron	126
Zinc	49.4
Manganese	38.1
Copper	9.6

### Phytochemical analysis

#### Total phenols, free polyphenols, tannins, and procyanidins content

**Fig. 1:**
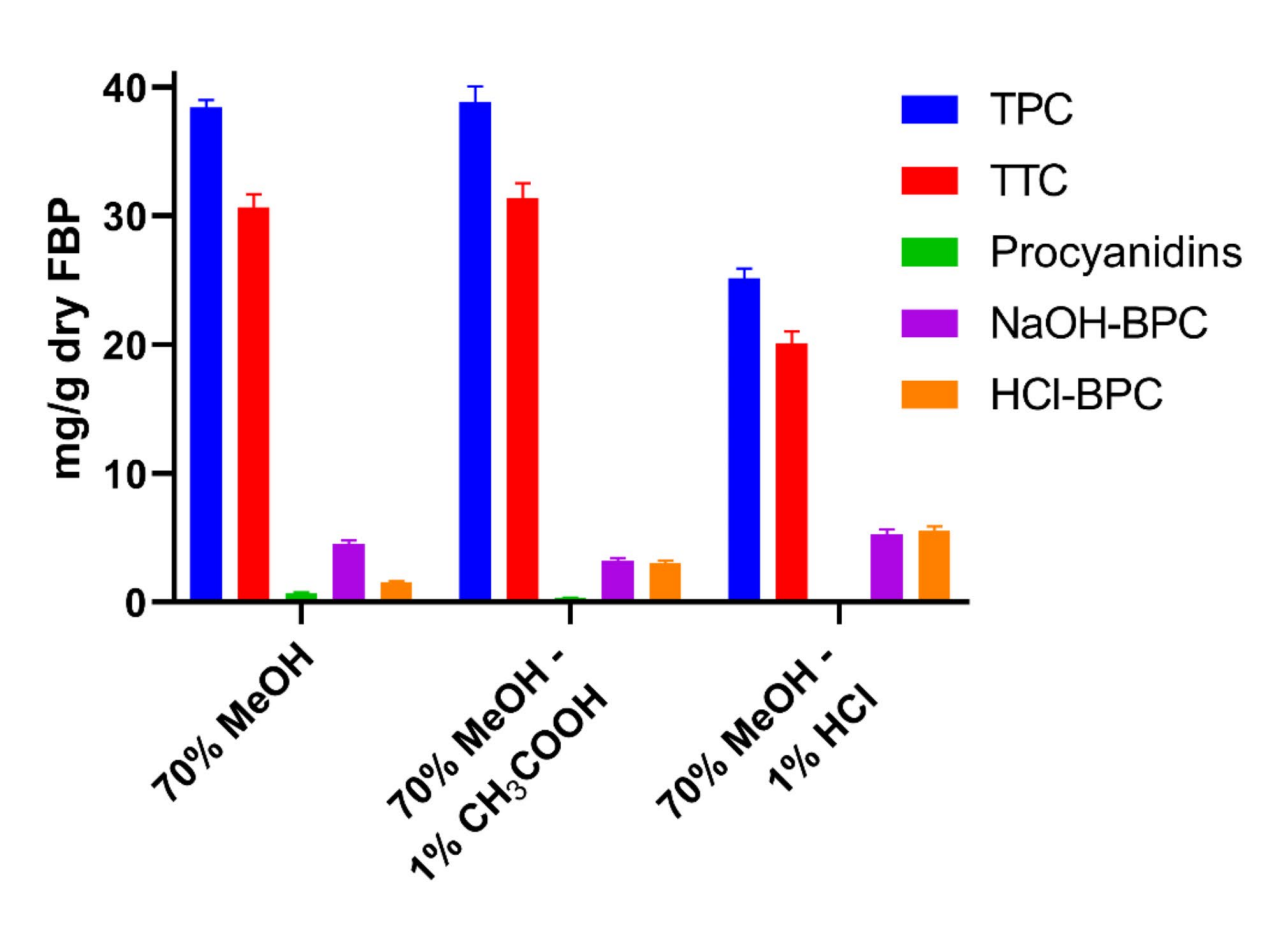
Quantitative analysis of phytochemicals in different extracts of faba bean pods. *TPC* total extractable polyphenols, *TTC* total extractable tannins, *NaOH-BPC* alkaline hydrolysed bound polyphenols, *HCl-BPC* acid hydrolysed bound polyphenols.

The quantitative analyses were carried out to determine the total content of phenols, bound polyphenols, free polyphenols, tannins, and procyanidins in the extracts from FBP. The highest content of phenolic compounds (TPC), 38.43 ± 0.59 and 38.87 ± 1.21 mg GAE/g dry FBP, was shown in the 70% MeOH (FBPM) and 70% MeOH − 1% acetic acid extracts. This included tannins at 30.67 ± 1.03 and 31.34 ± 1.21 mg GAE/g dry FBP, and procyanidins 0.66 ± 0.13 and 0.35 ± 0.02 mg procyanidin B1/g as presented in Fig. 1. The lowest recovery of the TPC and tannins was observed in the 70% MeOH – 1% HCl extract (25.17 ± 0.75 and 20.09 ± 0.95 mg GAE/g dry FBP, respectively). In addition, no procyanidins were quantified using this solvent. The 70% MeOH-1% HCl extract had the highest NaOH-BPC and HCl-BPC contents, with values of 5.23 ± 0.43 mg BPC/g FBP and 5.25 ± 0.33 mg BPC/g FBP, respectively (Fig. 1). In comparison, the 70% MeOH extract had lower HCl-BPC content (1.56 ± 0.08 mg BPC/g FBP), but a relatively high NaOH-BPC level (4.51 ± 0.28 mg BPC/g FBP). The 70% MeOH-1% CH₃COOH extract showed similar levels of NaOH-BPC and HCl-BPC (3.23 ± 0.19 mg BPC/g FBP and 3.02 ± 0.22 mg BPC/g FBP) (Fig. 1). Overall, the difference in polyphenols recovery between 70% MeOH-1% HCl and rest solvents was ~ 10% (Fig. 1), however the differences were statistically not significant. Given the potential health and environmental hazards of acetic and hydrochloric acids used in the extraction process, the 70% MeOH extract (FBPM) was selected for further analysis.

#### LC-MS

**Fig. 2:**
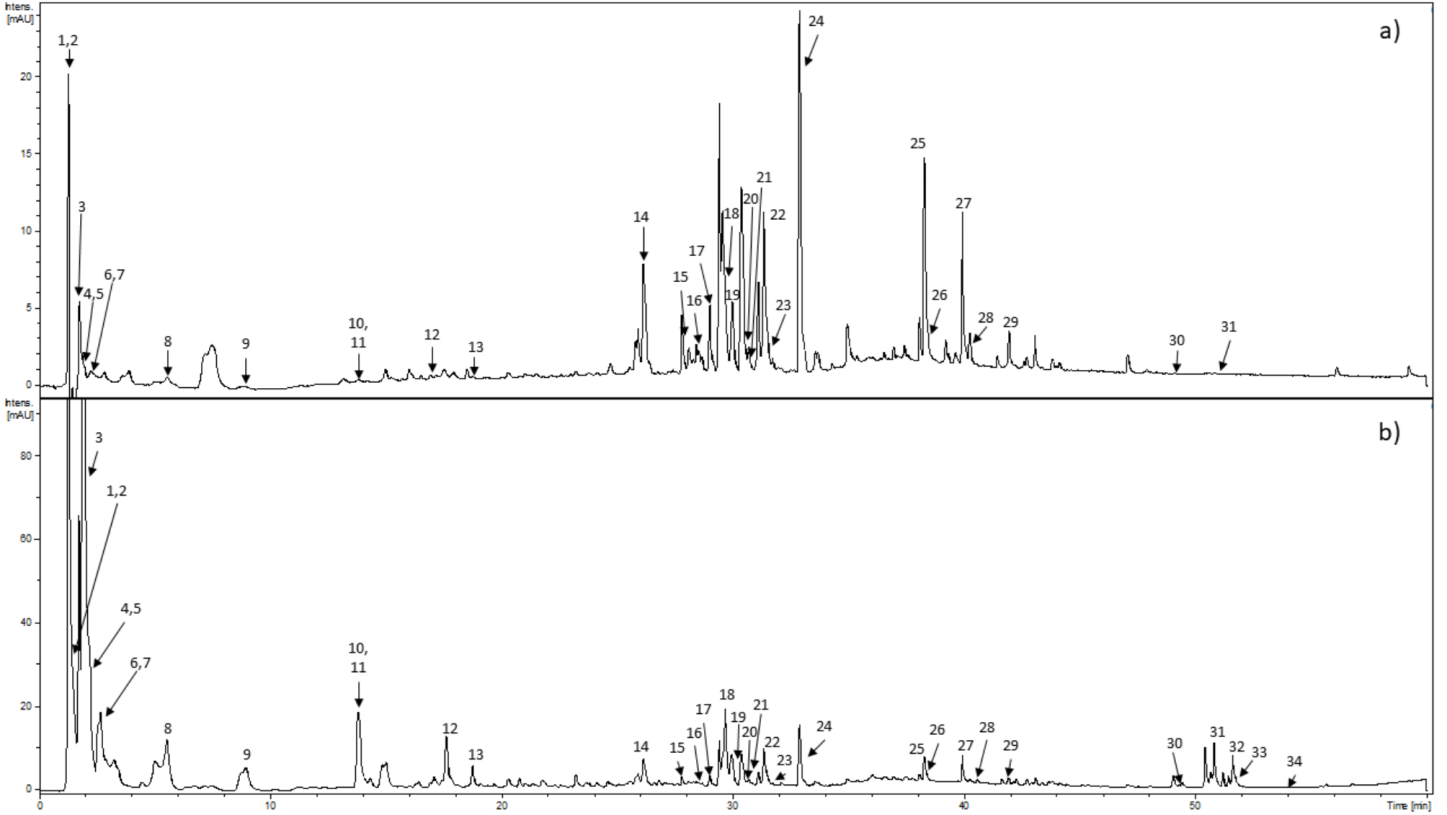
HPLC-DAD-Ms profile of FBPM was recorded in (**a**) 350 nm and (**b**) 280 nm. Compounds are named according to Table 2.

The comprehensive evaluation of the chemical composition of the FBPM extract was established using the UHPLC-DAD-MS^n^ method. The characterization of compounds was performed based on the UV–Vis maxima observed with a DAD detector and MS and MS/MS spectra recorded with an ion trap mass spectrometer. The analysis revealed the presence of 34 major compounds (Fig. 2a), and half of them remain unidentified (Table 2). In total, 16 compounds were identified. Compound **4** was found in the MS (+) spectrum at 198 m/z and fragmentation to m/z 181 and 152 as levodopa, which is used as a ‘gold standard’ of Parkinson’s therapy^16^. Vestitol (compound **9**) was preliminary defined for the first time in faba in MS (-) at 271 m/z followed by fragmentation to 179, 191, 195, 217, 235, 253^17^. Piscidic acid (compound **11**), 3’-O-methyl-(3’,4’-dihydroxybenzyl tartaric acid) (compound **12**), and hydroxyeucomic acid, (compound **13**) were identified based on available literature data^18,19^. The most abundant group of phytochemicals identified belonged to flavonoids: compounds (**15**, **16**, **17**, **21**, **22**) were classified as quercetin derivatives, while compounds (**18**, **19**, **20**, **24**, **25**, **26**) were identified as kaempferol derivatives (Fig. 2b; Table 2). Next, compounds **15–17**, **21**, and **22** were tentatively identified as quercetin derivatives^19–21^ based on the observation of an aglycone fragment ion at m/z 301 in the MS3 spectrum, indicative of the loss of a sugar moiety^22^, and the characteristic UV-Vis absorption maxima of the aglycone^23^. Two of them (compounds **15** and **16**) could be quercetin rhamnohexoside by the fragment ion 447 m/z in the MS2 (-), and quercetin rhamnopentoside was found as a compound **17**^19^. Compound **18** with MS (-) ion of m/z 593 was assigned as kaempferol rhamnopentoside and showed a loss of rhamnose residue in the MS2 (-) and fragment ion of kaempferol (285 m/z)^21,24^. Kaempferol dirhamnoside (compound **19**) was eluted at 30.1 min, and this non-acylated triglycoside had the deprotonated molecular ion at 709 m/z and fragmentation in MS2 (-) (563 m/z), MS3 (417, 285 m/z)^19^. Another flavonols glycoside was characterized by the loss of 146 m/z (rhamnosyl moiety) in MS2 (-) and MS3 and tarrying deprotonated molecular ion of quercetin (593 m/z) in the MS (-) (compound 21)^21^ along with pseudomolecular ion at 432 m/z in the MS (-), which produced kaempferol fragment ion (MS2 (-) = 285 m/z) (compound **25**)^19^. Additionally, the presence of kaempferol arabinopyranoside (compound **20**) in the extract was detected (MS (-) at m/z 563, and MS2 (-) at m/z 505, 430, 417, and 285^19,21^. Thus, compound **22** was unequivocally identified as quercetin acetylgalactorhamnoside, and its presence was confirmed in published data^19^. The molecular ion on MS (-) at 636 m/z has shown the fragments ion at 285 m/z (kaempferol aglycone) after losing a fragment of 204 m/z marked as a kaempferol acetylgalactorhamnoside (compound **24**)^21^. Compound **26** was assigned as kaempferol in agreement with the MS data and the fragmentation pattern^25^.

**Table 2:** UV–Vis and MS data of compounds identified in the 70% MeOH extract of faba bean pods.

Peak number	Identification	Retention time [min]	UV–Vis max [nm]	MS ions (−), [m/z]	MS2 (−), [m/z]	MS3, [m/z]	MS ions (+), [m/z]	MS2 (+), [m/z]	Citations
1	Unknown	1.5	225, 281	439	**421**, **341**, 327, 277, **259**, 179	–	–	–	–
2	Unknown	1.5	225, 281	719	379, **377**, 358, 341	–	–	–	–
3	Unknown	1.9	225, 282	401	354, 339, 310, 211, 197, **189**, 127	–	–	–	–
4	Levodopa (L-3,4-dihydroxyphenylalanin)	2.1	225, 280	196, 393	–	–	198	181, 152	^26^
5	Unknown	2.1	225, 280	415	218	–			–
6	Unknown	2.5	225, 282	405	**387**, 213, **191**, 173	–	407	389, 371, 257, 239, 215, 148	–
7	Unknown	2.5	225, 282	619	427	–			–
8	Unknown	5.7	232, 286	324	**306**, 280, **262**, 236, 192	–	326	**308**, **280**, **262**, 239, **216**	–
9	Vestitol	9.0	219, 280	271	253, 235, 217, 195, 191, 179	–	295a	277	^17^
10	Unknown	13.9	220, 274	533	**457**, **277**, **255**, 193, **165**	–	535	–	–
11	Piscidic acid	13.9	220, 274	255	237, 209, **193**,** 165**, **147**	–	279a	239	^18,27^
12	3ʹ-*O*-methyl(3ʹ,4ʹ-dihydroxybenzyl tartaric acid)- (3ʹ-*O*-methylfukiic acid)	17.8	225, 279	285	267, 239, **223**, 209, **195**, 163	–	309a	290, 272, 247, **135**	^19^
13	Hydroxyeucomic acid	18.9	222, 278	255	237, 219, 165, 139, 123	–	279a	278, 243	^19^
14	Unknown	26.3	296, 325	–	–	–	319	301, 274, 250, 229, 185, **157**	
15	Quercetin rhamnohexoside I	27.9	214, 266, 288	609	585, 539, **463**,** 447**, 397, 302, 301	301	–	–	^19,20^
16	Quercetin rhamnohexoside II	28.5	214, 288, 325	609	589, 576, 562, 493, **463**,** 447**, 299, 301	301	–	–	^19,21^
17	Quercetin rhamnopentoside	29.1	214, 253, 350	579	511, 446, **433**, 301	300	581	–	^19^
18	Kaempferol rhamnopentoside	29.6	214, 265, 316, 350	593	525, **447**, 431, 285, 255, 189	327, 284, 255, 151	595	–	^21,24^
19	Kaempferol dirhamnoside	30.1	214, 268, 313	709	663, 565, **563**, 430, 285	473, 417, 399, 381, 326, 310, 284, 255, 225, 213, 207, 179	711	–	^21^
20	Kaempferol arabinopyrano-side	30.6	214, 266, 344	563	505, **430**,** 417**,** 285**	–	565	–	^19,21^
21	Quercetin rhamnoside	30.8	214, 266, 341	593	546, 525, **447**, 446, 301, 285	301	617a	–	^19,21^
22	Quercetin acetylgalacto-rhamnoside	31.3	214, 264, 350	651	609, **505**, 447, 446, 301, 217	463, 445, 367, 343, 300, 271, 259, 232, 221, 217	653	–	^19^
23	Unknown	31.6	214, 296, 326	601	**564**,** 531**, 465, 269	–	–	–	–
24	Kaempferol acetylgalacto-rhamnoside	33.1	214, 265, 350	636	577, **489**, 430, 285	–	637	–	^21^
25	Kaempferol rhamnoside	38.2	217, 266, 364	432	363, **285**,** 151**	–	–	–	^19^
26	Kaempferol	38.5	217, 267, 345	285	211	–	287	269, 267, 240, 228, 227, 159, **135**, 122, 105	^28^
27	Unknown	40.0	217, 267, 336	269	211	–	271	145, 81	–
28	Unknown	40.6	219, 283	330	**311**, 310, 293, **229**, 225, 209, 202, 199, 183, 171	–	353a	336, **253**, 251, 235	–
29	Unknown	42.1	219, 265, 345	299	231, 229	–	301	–	–
30	Unknown	49.3	220, 289	593	547, 525, 477, **413**, 315, **277**, 241	–	–	–	–
31	Unknown	50.9	220, 279	294	238, **225**, 209, **185**, 167, 163, 149, 139	**–**	295	**277**, 259, 231, 159, 147, 131	**-**
32	Unknown	51.6	220, 274	595	527, **415**, 315, **279**, 241	–	–	–	–
33	Unknown	51.8	220, 275	–	–	–	579	301	–
34	Unknown	53.7	220, 275	579	546, **511**, **443**, 307, 229, 225	–	–	–	–

a—[M + Na]; in bold—the most abundant ion in the recorded spectrum.

### Interaction with porcine digestive enzymes

**Fig. 3:**
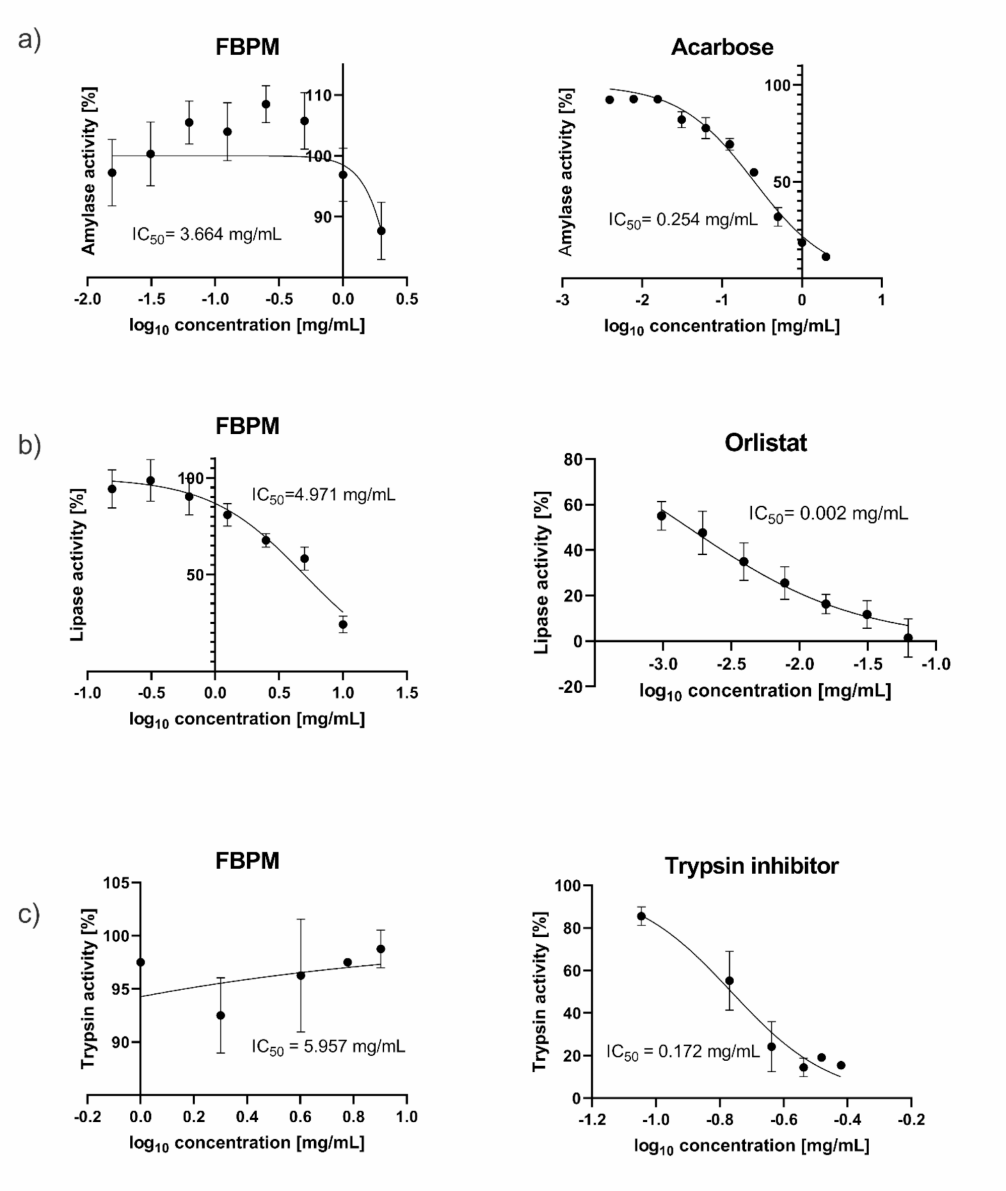
The effect of FBPM extract and inhibitors on digestive enzymes activity: (**a**) α-amylase; (**b**) lipase; (**c**) trypsin.

To assess the suppressive effects of the FBPM extract on piglets digestive enzymes, including α-amylase, lipase, and trypsin, we employed both fluorometric and spectrophotometric techniques. Our findings revealed that the FBPM extract exhibited minimal activity towards all tested enzymes (Fig. 3). In the α-amylase assay, the FBPM extract showed a half maximal inhibitory concentration (IC_50_) of 3.664 mg/mL (Fig. 3a), in addition in the lipase assay, the IC_50_ was calculated as 4.971 mg/mL (Fig. 3b). Finally, for trypsin inhibition, the FBPM extract presented an IC_50_ of 5.957 mg/mL (Fig. 3c). In all three assays, the positive controls – acarbose for α-amylase (IC_50_ = 0.254 mg/mL), orlistat for lipase (IC_50_ = 0.002 mg/mL), and trypsin inhibitor from the chicken egg for trypsin (IC_50_ = 0.172 mg/mL) – caused a significant reduction in enzyme activity when compared with the uninhibited control (Fig. 3). This is as expected and provided validation of the assay methods and confirmed the suitability of the assays for their intended purpose.

### Antibacterial activity

**Fig. 4:**
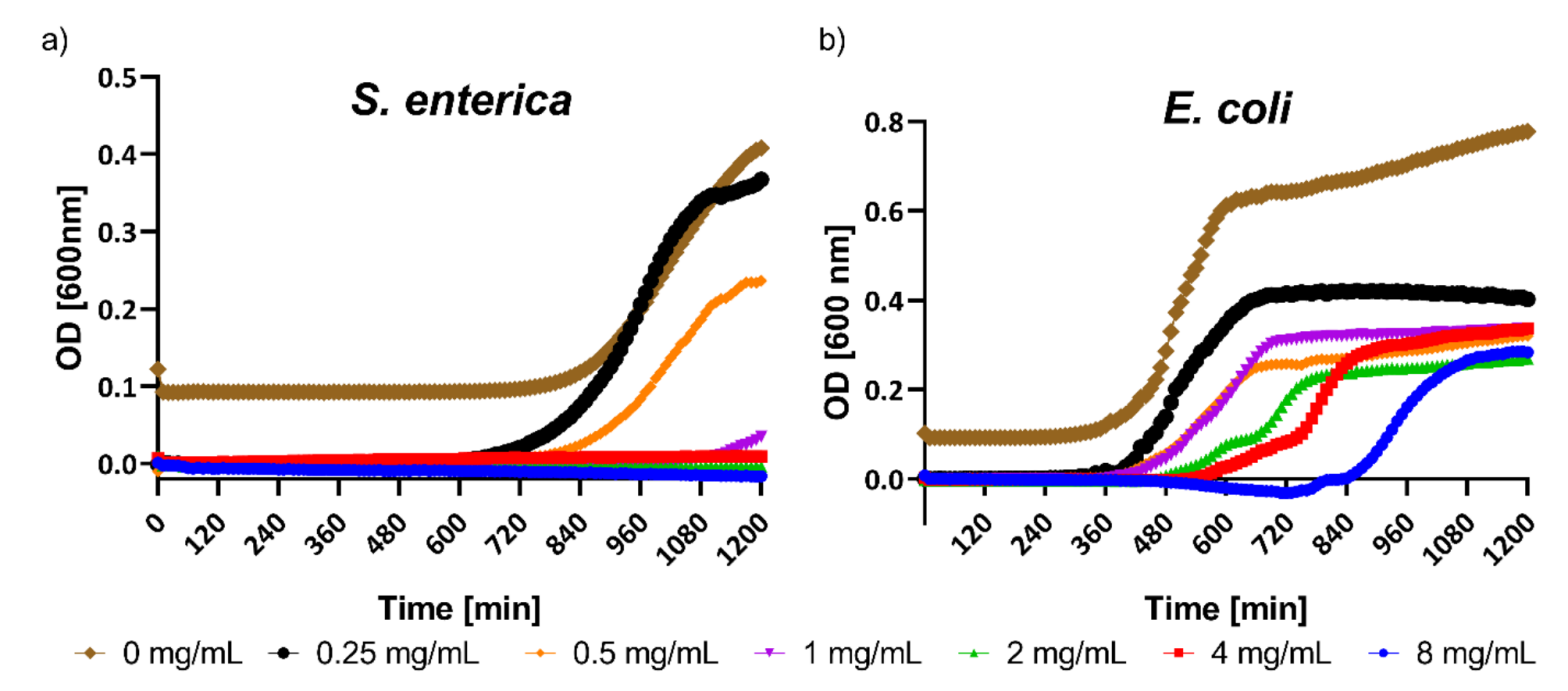
Growth inhibition of (**a**) *S. enterica* and (**b**) *E. coli* after the treatment with FBPM extract. For *S. enterica*, the differences between growth control and 1, 2, 4, and 8 mg/mL after overnight incubation are statistically significant with *p* < 0.01. For other concentrations, the differences between the growth control and the respective concentrations are not significant with *p* > 0.05. For *E. coli* statistically significant (*p* < 0.05) delay in lag time between the growth control and treated samples was observed starting from 560 min.

**Fig. 5:**
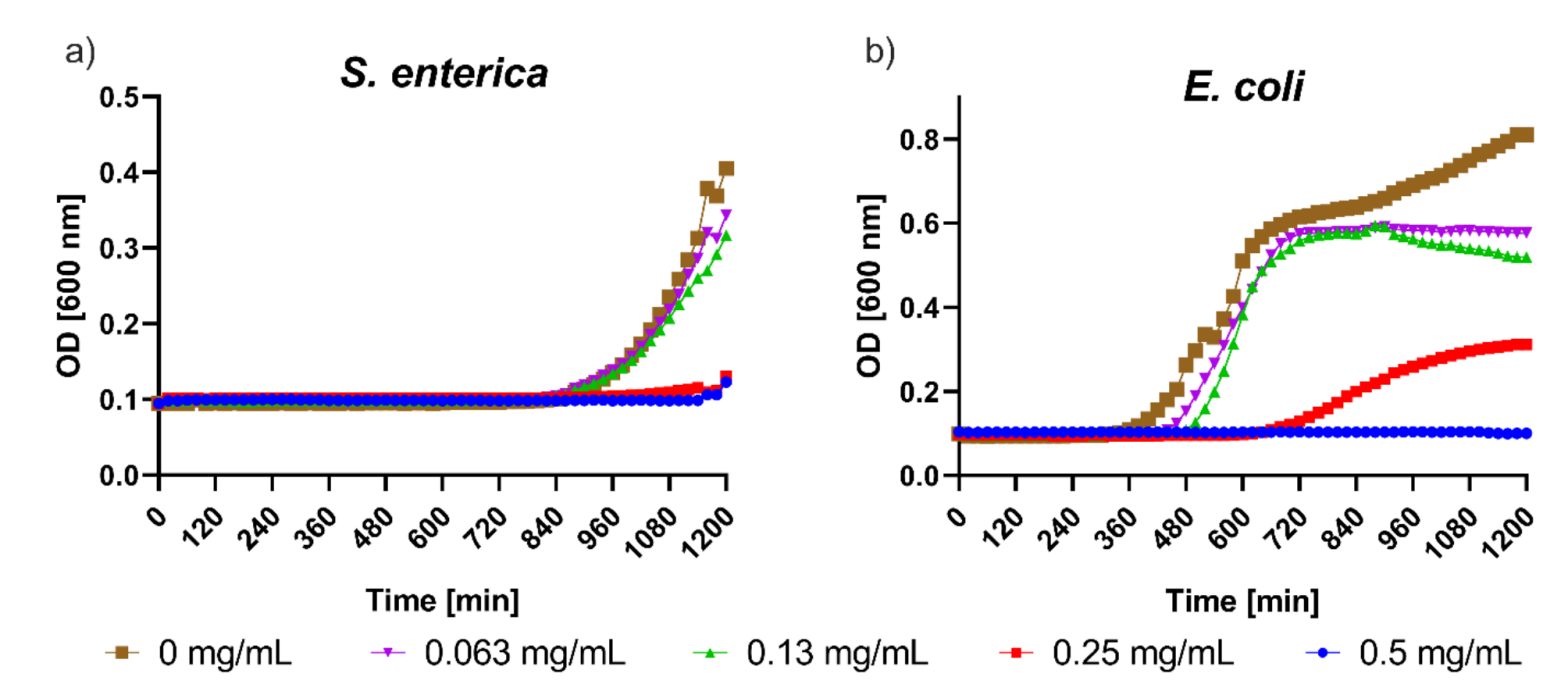
Effect of piscidic acid on the growth of (**a**) *S. enterica* and (**b**) *E. coli*. For *S. enterica*, the differences between 0 mg/mL (growth control) vs. 0.25 and 0.5 mg/mL are statistically significant with *p* < 0.01. For *E. coli*, the difference between growth control and 0.5 mg/mL is statistically significant with *p* < 0.01. For other concentrations, the differences between the growth control and the respective concentrations are not significant with *p* > 0.05.

We evaluated the potential of the FBPM to inhibit the growth of *S. enterica* and *E. coli*; two of the most common bacterial agents involved in gastrointestinal infection. We cultured bacteria in a BHI medium in the presence of FBPM extract in a concentration range of 0.25 ─ 8 mg/mL. As presented in Fig. 4a, we observed a complete inhibition of *S. enterica* visible growth at a concentration of the FBPM extract 1 ─ 8 mg/mL. Therefore, the minimal inhibitory concentration (MIC) was determined as 1 mg/mL. The differences between the growth control and concentrations of 1, 2, 4, and 8 mg/mL are statistically significant (*p* < 0.01) after the overnight incubation. At other concentrations, the differences compared to the growth control are not significant (*p* > 0.05). However, for *E. coli* we observed a concentration-dependent growth inhibition, seen as a prolonged lag time (Fig. 4b). The duration of the lag phase increases with an increase in the concentration of the extract. A statistically significant (*p* < 0.05) delay in lag time between the growth control and the treated samples was observed starting from 560 min. Next, we sought that the observed antimicrobial effect might be attributed to the presence of piscidic acid in the extract. Therefore, we tested the effect of piscidic acid on the growth of *S. enterica* and *E. coli* in concentrations of 0.063 mg/mL – 0.5 mg/mL. We found complete growth inhibition of *S. enterica* in concentrations of 0.25 and 0.5 mg/mL (Fig. 5a), determining the MIC to be 0.25 mg/mL. We also found that piscidic acid inhibited the growth of *E. coli* with MIC = 0.5 mg/mL (Fig. 5b). The differences between the growth control (0 mg/mL) and 0.25 mg/mL for *S. enterica* and 0.5 mg/mL for *E. coli* are statistically significant (*p* < 0.01). Differences between the growth control and other concentrations are not statistically significant (*p* > 0.05).

As reference controls, we used commonly utilized antibiotics: ciprofloxacin for *S. enterica* (MIC = 0.06 µg/mL) (Figure S1) and kanamycin for *E. coli* (MIC = 12.5 µg/mL) (Figure S2). Furthermore, the application of methanol at concentrations up to 3%, which corresponds to those in the tested samples with the extract, did not impact bacterial growth (Figure S3). Therefore, we concluded that the observed inhibitory effect was due to the plant extract activity.

### Cytotoxicity study

**Fig. 6:**
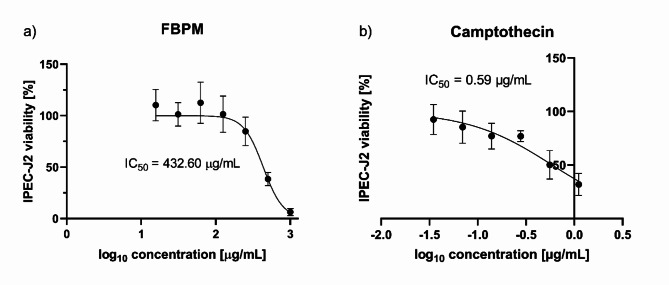
Viability of IPEC-J2 cells after 24 h of incubation with (**a**) FBP extract, (**b**) camptothecin determined by the MTT assay. For FBPM the difference between 0 vs. 250 µg/mL, 0 vs. 500 µg/mL, and 0 vs. 1000 µg/mL are statistically significant with *p* < 0.01. For camptothecin the difference between 0 vs. 0.56 µg/mL, and 0 vs. 1.11 µg/mL are statistically significant with *p* < 0.01.

To determine the potential cytotoxic effect of the FBPM extract on the porcine intestinal IPEC-J2 cells, the MTT assay was used. The FBPM was tested in a concentration range of 15 to 1000 µg/mL, and it exhibited minor cytotoxicity with a half-maximal inhibitory concentration (IC_50_) = 432.6 µg/mL (Fig. 6a). The positive control - camptothecin - showed high toxicity with an IC_50_ = 0.59 µg/mL (Fig. 6b). No significant impact on cell viability was observed in the negative control treated with 0.35% DMSO.

## Discussion

The focus of our study was to assess the chemical constituents of the FBP cultivar “Bizon”, which included a comprehensive analysis of its phytochemical and nutritional composition. In addition, our investigation included the evaluation of the effects on porcine digestive enzymes, antibacterial activity, and cytotoxicity against porcine intestinal epithelial cells associated with FBP. The cultivar “Bizon” is one of the most popular cultivars grown in Poland, making industrial by-products easily accessible and available in large amounts. Besides, “Bizon” was first registered in 1955, and since then, it has not been subjected to breeding strategies aiming at the improvement of crop performance or nutritional values.

As mentioned in the literature review, the main components of the pods are fibres^2^, which was confirmed in the present study. While the protein content is relatively high in our study (144 g/kg, which converts to 14.4%), it remains lower when compared to other species, which typically exhibit protein levels ranging between 26.2% and 32.8%. Our findings show that FBP contains primarily insoluble fibre (328 g/kg = 32.8%) with smaller quantities of soluble fibre (84.6 g/kg = 8.46%), in line with previously reported ranges (10.70–15.96% and 0.55–1.06%, respectively)^3^. Although dietary fibre is not metabolized by mammalian enzymes, it has beneficial effects on health due to the maintenance of healthy gut microbiota and the gut-associated immune system, as well as being a source of short-chain fatty acids^29–31^. The mineral content of FBP is comparable to previously published data^3^; for example, in our case, the zinc concentration measured was 49.39 mg/kg, which is equivalent to 4.94 mg per 100 g, and the study by Labba et al. showed that zinc concentrations ranging from 0.9 to 5 mg per 100 g for different species^3^. The content is also similar to those profiled in seeds or beans^32^. These findings suggest that FBP can serve as a nutritionally valuable alternative source of protein, fibre, and essential trace elements, potentially offering cost-effective and sustainable feed additives in agricultural systems.

The chemical composition of the extract prepared from the plant material is a critical factor in determining its biological activity. To prepare extracts from FBP, we used methanol as a base solvent. This aimed to prevent degradation or undesirable oxidation of phenolic compounds, hence a decrease in phenolic yields and quality of plant material^33^. To accelerate a mass transfer of phenolic compounds, we also tested methanol with the addition of either acetic or hydrochloric acid. This was intended to result in higher TPC if a significant concentration of complex polyphenols, such as proanthocyanidins, were present in the FBP^34^. However, that was not the case. The presence of neither acid improved TPC recovery, suggesting low amounts of proanthocyanidins, which we further confirmed with HPLC analysis. The highest TPC was obtained through extraction with 70% MeOH, indicating that the phenolic compounds in FBP were of high polarity, including flavonoids as glycosides and less polar aglycones^35^.

So far, most studies have focused on beans and harvest residues, such as stems and leaves, while the pods have received less consideration in phytochemical analyses. Therefore, the chemical content of pods is not very well documented in the literature^2^. Chaieb et al. reported that the phytochemical composition depends on the stage and the parts of the plant in the study of thirteen genotypes of *Vicia faba* L^36^. Considering these aspects, only two published reports on the TPC in pods cultivated and processed under similar condition, revealed that TPC ranged from 56.93 to 149.21 mg GAE/g in ethanolic extracts and 115.21 mg GAE/g in methanolic extracts^20,36^. In our study, the highest content of phenolic compounds was found in the 70% MeOH and 70% MeOH − 1% acetic acid extracts, yielding 38.43 ± 0.59 and 38.87 ± 1.21 mg GAE/g, respectively. Although our results differ from the previously reported ranges, this discrepancy may be due to differences in the extraction methods used. It is noteworthy that the presence of phenolic compounds can vary significantly across various plant parts. Prior investigations have revealed that faba bean sprouts possess a total phenolic content ranging from 11 to 13.7 mg GAE/g of fresh weight^37,38^, and the amount of TPC in green seeds was determined to be 20.89–44.65 mg GAE/g of sample on a dry weight basis^39–41^. Higher phenolic content was observed in faba bean hulls (50.65 mg GAE/g of extract)^42^, while the flowers showed a TPC value of 93.20 mg GAE/g of dried plant^43^.

We next evaluated the chemical composition of the 70% MeOH extract using UHPLC-DAD-MS^n^, and found the presence of 34 major compounds. The findings of the current study support the previous studies on faba pods by Valente et al.^18^. as most of the identified compounds are specialized metabolites, such as phenolics, flavonoids and their derivatives, and L-DOPA – the precursor of dopamine. The dominant compounds were flavonoids, primarily glycosides of quercetin and kaempferol. Interestingly, we found vestitol (compound 9), which has been identified in *Vicia faba* L. for the first time. Potential anti-inflammatory activity was previously indicated for vesitol^17^; however, the content of this compound in FBP may be insignificant for expressing biological activity. Opposite to faba seeds, where primarily catechin and epicatechin derivatives were found^39^, pods and beans contain more quercetin and kaempferol derivatives^18,19^. On the other hand, we found several unknown compounds that still need to be identified and isolated. The above-mentioned results confirm that the FBP is a valuable source of diverse phytochemicals that can express interesting biological activities.

The prevalent view of faba beans in piglet nutrition is that they contain high levels of anti-nutritional factors, particularly tannins and protease inhibitors^32^. These compounds can reduce feed intake and impair the digestibility of key nutrients, such as protein and minerals. Consequently, piglets may exhibit slower growth rates and lower feed conversion efficiency^44,45^. As a result, the use of tannin-containing feed additives has been minimized in piglets diets. So far, no study specifically investigated the impact of anti-nutritional factors from FBP on piglets growth. Research on faba bean seeds or beans as a plant protein source shows no negative effects on piglets growth performance when included at levels of 20–25% in the diet^46,47^, even though beans and seeds are known to be rich in condensed tannins^20,39^. In the present study, qualitative analysis of the tested pods revealed no tannins or their derivatives, which further reduces their anti-nutritional properties. The results of the total tannin and total procyanidin content analyses conducted using colorimetric methods indeed indicated the presence of tannins. However, the precise analysis of the extract using LC-MS method did not show any compounds identified as tannins or any artifacts indicating their presence in native plant material. The previous reports of tannins presence in faba beans are mainly based on the results obtained by colorimetric methods, which, as seen in the present study, are not specific and can give false positive results. To our knowledge, no compound belonging to the group of tannins has been unambiguously found or isolated from *Vicia faba* L. or other species belonging to *Vicia sp*.

On the other hand, another study on beans and seeds demonstrated significant inhibition in the in vitro enzymatic activities of trypsin, amylase, and lipase^48,49^. Here, we report that FBP does not significantly affect the activity of any of these digestive enzymes in vitro. The variations in bioactivity and their anti-nutritional properties between different parts of faba bean plant are therefore attributed to differences in the composition of phytochemicals, including the accumulation of tannins^50^.

The chemical composition of the analysed extract, particularly the abundance of polyphenols, suggested promising antibacterial potential. The antimicrobial activity of phenolic compounds is well documented, and different mechanisms have been proposed^51,52^. In this context, adsorption of phenolic compounds to cell membranes and their interaction with enzymes and metal ions deprivation has been proposed as a mechanism of action of pure phenolic compounds such as gallic acid, vanillic acid, caffeic acid, rutin, and quercetin against *E. coli* and *Flavobacterium sp.*^53^ Our study supports evidence from previous observations that *Vicia faba* L. is a valuable source of antimicrobial compounds. For example, Mejri et al. reported the antimicrobial activity of different extracts from broad bean pods against various gram-positive and gram-negative bacteria as well as the yeast *C. albicans*, which were consistent with our findings^20^. Furthermore, antimicrobial screening of aqueous extract from flowers and ethanolic extracts from leaves showed inhibition of *E. coli* growth in a disk diffusion assay^54^. In a similar assay, Chaurasia et al. also showed inhibitory activity against *E. coli*^55^; however, the inhibition zones observed in this research were smaller than the ones observed by Peyvast and Khorsandi. Nevertheless, it shows that the faba bean pods can be used not only as a nutritional supplement but also as an alternative to antimicrobials in the prevention of gastrointestinal infections in farm animals.

Finally, we performed the cytotoxicity assay of the FBPM extract using a porcine intestinal cell line IPEC-J2. The FBPM showed neglected cytotoxicity (IC_50_ = 432.6 µg/mL), which preliminary proves its safety of potential usage as a feed supplement for piglets. Similar observations were reported for human embryonic kidney (HEK) 293 cells, and normal human breast cells MCF-10 A with IC_50_ > 500 µg/mL for both cell lines^56^. Moreover, Mejri et al. investigated the in vivo activity of orally administered methanol extract of faba bean pods in a mice model. They proved the potential of FBP methanol extract to exert antidiabetic effects as well as its beneficial impact on liver, and kidneys^20^. Altogether, these findings suggest that FBP could have potential to be safely applied as an effective feed additive in animals.

In conclusion, our study addresses the paradigm on the anti-nutritional properties of faba bean and put a new light on the potential utilization of FBP cultivar “Bizon” in the prevention of infections in piglets caused by *E. coli* and *S. enterica*. The FBP showed no adverse effects on porcine intestinal epithelial cell line, and relevant enzymes required for efficient digestion. While the findings presented in this manuscript originate from in vitro analyses, they can serve as an initial point for further studies on the development of novel, sustainable feed additives dedicated to piglet nutrition being alternatives to antibiotics. It is important to note, however, that the content of phytochemicals can vary quite strongly depending on origin, environmental conditions, time of harvest, and cultivars. Therefore, the determination of the structural characterization and the composition of anti-nutritional factors, such as tannins, before applying the pods as feed additives is a must.

## Methods

### Solvents and reagents

Acetonitrile, methanol, and formic acid for HPLC were purchased from Merck (Darmstadt, Germany). Water was purified with a Millipore Simplicity System (Bedford, MA, USA). Acetone, acetic acid, ethanol, hydrochloric acid, sodium hydroxide, diethyl ether, and ethyl acetate were obtained from POCh (Gliwice, Poland). Folin–Ciocalteu reagent, sodium carbonate, polyvinylpolypyrrolidone, 4-(Dimethylamino)cinnamaldehhyde, 4-Methylumbelliferyl oleate, Nα-benzoyl-L-arginine ethyl ester hydrochloride, sodium phosphate, porcine pancreas enzymes: trypsin (Cat. T0303), lipase (Cat. L3126), α-Amylase (Cat. A3176), and antibiotics (kanamycin, ciprofloxacin) were obtained from Sigma-Aldrich (Germany). Reference controls for enzyme inhibition assays: orlistat (Cat. PHR1445), trypsin inhibitor from chicken egg white (Cat. T9253), and acarbose (PHR1253) were purchased from Sigma-Aldrich (Germany). EnzChek™ Ultra Amylase Assay Kit was obtained from Invitrogen, USA. Chemical standards: gallic acid (Sigma, USA), piscidic acid (Aobious, USA), and procyanidin B1 (PhytoLab, Germany) were of HPLC grade.

### Plant material

Faba bean (*Vicia faba* L.*)* cultivar ‘Bizon’ (seeds obtained from PlantiCo Hodowla i Nasiennictwo Ogrodnicze Zielonki Sp. z o.o.) was planted in Umer, Świętokrzyskie, Poland. Pods were collected and peeled between July 5th and July 30th, 2022. The green pods were shade-dried until they reached a constant weight, ground into a powder using a blender, and stored in a sealed container in a dark place at room temperature. The plant material was confirmed anatomically and morphologically based on the Voucher specimen (No. FBPU2022), which has been deposited in the Department of Pharmaceutical Biology, Medical University of Warsaw, Poland.

### Nutrient analysis

The nutrient content in the air-dried faba bean pods was analysed according to the official standard methods of the Association of German Agricultural Analytics and Research Institutes (VDLUFA) (3.1 dry matter, 8.1 crude ash, 5.1.1 ether extract)^57^. The crude protein (4.1.2) was determined after the Dumas method according to the Macro N-determination with a Vario Max CN (V7.3.9 Elementar Anaylsesysteme GmbH, Hanau, Germany) (VDLUFA)^57^. Amylase-treated organic neutral detergent fibre, and acid detergent organic fibre were analysed with the determination of the residual ash in accordance with the VDLUFA method (6.5.1, 6.5.2)^57^ using the ANKOM 2000 Automated Fibre Analyzer (NY, USA). Crude fibre was performed with the same apparatus using the AOCS method (Ba 6a-05, AOCS 2017)^58^. Total dietary fibre, insoluble dietary fibre, and soluble dietary fibre were analysed with a commercial assay kit (Megazyme K-TDF, Megazyme, Ireland). Phosphorus was analysed according to the vanadate-molybdate method with a spectrophotometer at 436 nm (Ultospec 2100 pro, Amersham Biosciences Europe GmbH, Germany)^59^. Calcium, sodium, potassium, magnesium, iron, manganese, copper, and zinc were first dry-ashed and then measured with atomic absorption spectrometry (ContrAA 700, AnalyticJena AG, Jena, Germany)^57^.

### Extract preparation

Three extracts were prepared in the same way using different solvents. A portion of grounded plant material was mixed in a ratio of 1:10 (w/w) with one of the following solvent combinations: (1) H_2_O/methanol (30:70, v/v); (2) H_2_O/methanol/acetic acid (29:70:1, v/v) or (3) H_2_O/methanol/hydrochloric acid (29:70:1, v/v). The mixtures were then extracted using an ultrasonic bath (30 min at 40 °C). Subsequently, the extracts were centrifuged (2450 g, 5 min, room temperature), and supernatants were transferred to new tubes. These steps were repeated three times, after the supernatants were combined and filtered with a paper filter. The solvents were evaporated using a rotary evaporator at 40 °C. The final step involved freeze-drying (48 h, vacuum < 1 mBar, -80 °C) of the supernatants and pellets. The dry supernatants constituted ~ 30% by weight of the used plant material.

### Phytochemical analysis

#### Total extractable polyphenol content

Total extractable polyphenol content (TPC) was determined using the colorimetric Folin-Ciocalteu assay^60^. The extracts were dissolved in H_2_O/methanol (50:50, v/v) in a concentration of 1 mg/mL. 40 µL of each solution was added to the 96-well plate and mixed with 105 µL of 10% Folin-Ciocalteu reagent (Sigma, USA) and 85 µL of 1 M Na_2_CO_3_. The plate was incubated at 45 °C for 15 min, and the absorbance was recorded at 765 nm using a microplate reader (Agilent BioTek Synergy H4, USA). Gallic acid was used as a standard, and results were expressed as mg gallic acid equivalents per g dry FBP (mg GAE/g dry FBP) from the calibration curve. Results are presented as means ± SD from six independent experiments.

#### Total extractable tannin content

Modification of the Folin-Ciocalteu method using polyvinylpolypyrrolidone (PVPP, Sigma USA) was used to verify the amount of free polyphenols^61^. The test samples were prepared in deionized water (2 mg/mL), vortexed with a 10% PVPP solution, and cooled for 15 min at 4 °C. Next, mixtures were centrifuged (3000 g, 10 min at 4 °C), and the collected supernatants were mixed with methanol (1:1). 40 µL of the samples were added to the plate and mixed with 105 µL of 10% Folin-Ciocalteu reagent solution (Sigma, USA) and 85 µL of 1 M Na_2_CO_3_. The plate was incubated at 45 °C for 15 min, and the absorbance was recorded at 765 nm using a microplate reader (Agilent BioTek Synergy H4, USA). The total extractable tannin content (TTC) was calculated by subtracting the amount of free polyphenols from the TPC. The results were reported as mg GAE/g dry FBP and expressed as the means ± SD from six independent experiments.

#### Total procyanidin content

The 4-(Dimethylamino)cinnamaldehhyde (DMAC) colorimetric method^62^ was used for the estimation of the aggregate amount of proanthocyanidins in the extracts. The extracts (1 mg/mL) were prepared in acetone/acetic acid (99.5:0.5, v/v), agitated in an orbital shaker for 60 min, and centrifuged (2000 g, 20 °C for 10 min). A DMAC solution was freshly prepared in an ethanol/hydrochloric acid mixture (68:4.5, v/v). 70 µL of the supernatants were added to the 96-well plate and mixed with 210 µL of the DMAC solution. The absorbance of the reaction mixtures was measured at 640 nm (every minute for 30 min) using a microplate reader (Agilent BioTek Synergy H4, USA), and the maximum absorbance readings were used for calculation of the amount of proanthocyanins in the extracts. Procyanidin B1 was used to make a calibration curve. Results are expressed as means ± SD from six independent experiments.

#### Bound polyphenol content (BPC)

The resulting freeze-dried pellets, obtained after extract preparation, were used to separate bound polyphenols according to the method by Peng et al.^63^ with modifications. First, the bound phenolics obtained by alkaline hydrolysis (NaOH-BPC) were hydrolysed with 2 M NaOH at a ratio of 1:15 (w/w) relative to the initial mass of plant material. The mixture was placed in an ultrasonic bath for 15 min at room temperature, followed by 24 h in an orbital shaker. Next, the pH of the solution was adjusted to 2, and a liquid-liquid extraction^64^ using a diethyl ether/ethyl acetate mixture (DE/EA, 1:1) as a solvent was performed. The pellets were re-extracted three times, and NaOH-BPC supernatants were combined and evaporated at room temperature.

The remaining residues after NaOH-BPC were acid hydrolysed (HCl-BPC) with 2 M HCl (in a volume equal to 2 M NaOH); the solution was heated in a water bath (85 °C for 1 h), and allowed to cool before undergoing the liquid-liquid extraction (DE/EA, 1:1). The pellets were re-extracted three times, and HCl-BPC supernatants were combined and evaporated at room temperature and subjected to the colorimetric Folin-Ciocalteu assay as described in Method TPC. Results are expressed as means ± SD from three independent experiments.

#### LC-MS

The UHPLC-DAD-MS^n^ analysis was performed using an Ultimate-3000 RS system (Dionex, Leipzig, Germany) with a DAD detector and splitless connection with an AmaZon SL ion trap mass spectrometer with an ESI interface (Bruker Daltonik GmbH, Bremen, Germany). UV–Vis spectra were obtained over the range of 200–450 nm. The following parameters of the MS unit were used: nebulizer pressure (40 psi, drying gas flow rate: 9 L/min, nitrogen gas temperature 134 °C), and capillary voltage (4.5 kV). The mass spectra were registered by scanning from 70 m/z to 2200 m/z. The mobile phase (A) was H_2_O/formic acid (100:0.1, *v/v*), and the mobile phase (B) was acetonitrile/formic acid (100:0.1, *v/v*). The gradient programs and the flow rates were respectively 0–30 min 1–20% B, 30–40 min 20–50% B, 40–50 min 50–75% B, 50–60 min 75–100% B and 0.3 mL/min. Separation of compounds in the extract was on column Kinetex XB-C18 (Phenomenex, Torrance, California, USA, 150 mm × 3.0 mm × 2.6 μm). The column oven temperature was set to 25 °C. The dry extract (FBPM) and standards were dissolved in an H_2_O/methanol/formic acid (30/70/0.2, *v/v*) at a concentration 10 mg/mL and filtered through a 0.45 μm PVDF syringe filter prior to chromatographic screening. The injection volume was 3 µL^65^.

### Digestive enzymes inhibitory activity

#### α-amylase assay

The dose-dependent porcine α-amylase inhibitory activity of the extract was evaluated using an EnzChek™ Ultra Amylase Assay Kit according to the manufacturer’s instructions. The amount of fluorescent substance released from starch substrates was determined by measuring fluorescence (excitation at 485 nm and detection at 535 nm) with reference to an experimentally derived standard curve. Acarbose was used as a control. Based on the percentage of inhibition of α-amylase activity, half maximal inhibitory concentrations (IC_50_) were calculated both for extract and acarbose. Results are expressed as means ± SD from three independent experiments.

#### Lipase assay

The modification of pancreatic lipase inhibition assay using 4-Methylumbelliferyl oleate (4-MUO) as a substrate was used^66^. The substrate 4-MUO (0.5 mM) and the enzyme were prepared in 50 mM Tris-HCl buffer (pH 8), and the extract was dissolved in 70% methanol at a concentration of 10 mg/mL. Orlistat, also dissolved in 70% methanol, was used as a control inhibitor (0.0625 mg/mL). Serial dilutions of the extract and orlistat solutions were applied to a 96-well plate in a volume of 100 µL and mixed with 50 µL of the substrate solution. After a 10-minute incubation at 37 °C, 50 µL of the pancreatic lipase solution was added to the plate. The positive control wells included 70% methanol, the substrate, and enzyme solutions, while negative control included the buffer and substrate solutions. Fluorescence spectra were measured at an excitation of 320 nm and emission of 455 nm (every 5 min, with a shaker at 37 °C). Then, IC_50_ were calculated for the test sample and the inhibitor. Results are expressed as means ± SD from three independent experiments.

#### Trypsin assay

The assay was performed according to the protocol provided by Sigma-Aldrich: Enzymatic Assay of Trypsin Inhibitor^67^. Briefly, the trypsin inhibitor or test sample was incubated with 0.05 mg/mL of trypsin from porcine pancreas in 1 mM HCl at 25 °C for 5 min. The reaction was initiated by the addition of the substrate 0.25 mM BAEE (Nα-benzoyl-L-arginine ethyl ester hydrochloride) dissolved in 63 mM sodium phosphate. Residual trypsin activity was measured by monitoring the change in absorbance at 253 nm in 1-minute intervals for 5 min using a spectrophotometer (Shimadzu UV-160 A, Japan). The final concentrations in a reaction were as follows: 63 mM sodium phosphate, 0.23 mM BAEE, 0.002 mM HCl, 0.005 mg trypsin, and 0–8 mg of faba bean pods extract. As a control, trypsin inhibitor from chicken egg white (Cat. T9253, Sigma-Aldrich, USA) was used in concentrations 0–0.5 mg/mL. The percentage inhibition was calculated by using the following formula: % Inhibition = (ΔA_230nm_ control - ΔA_230nm_ test) * 100 / (ΔA_230nm_ control - A_230nm_ blank). The IC_50_ value was defined as the concentration of the compound where percent inhibition is equal to 50 and was the mean from at least two independent experiments.

### Antibacterial activity

*Escherichia coli* ATCC^®^ 25922 and *Salmonella enterica* subsp. *enterica* serovar Typhimurium LT2-R^68^ were used to determine the antibacterial activity of FBPM extract. To prepare the inoculum, all strains were grown overnight in BHI medium (Roth, Karlsruhe, Germany) at 37 °C with shaking. Overnight cultures were diluted 1:99 in a fresh BHI and incubated at 37 °C with shaking to the late exponential phase. FBP stock solution was prepared in 70% MeOH and sterilized by filtration through 0.22 μm PVDF syringe filters prior to the experiment. Aliquots of the cell suspensions were added to the wells of sterile 96-well plates containing dilutions of the tested compounds, yielding a final concentration of 10^4^ CFU/mL. Plates were sealed with adhesive film and incubated at 37 °C in a microplate reader (Agilent BioTek Synergy H4, USA) for 20 h. During incubation, the optical density at 600 nm (OD600) of the culture in each well was measured at 10-minute intervals. Before each measurement, the plate was briefly shaken to suspend the cells. For the determination of lag time (λ [min]), the lag-exponential (λL) method was used^69^. In all experiments, either ciprofloxacin (Polpharma, Poland) or kanamycin (Sigma-Aldrich, USA) was applied as a positive control. Each experiment was performed in three independent biological replicates. For the evaluation of statistical significance, the two-way analysis of variance test following multiple comparisons was used. A probability value of *p* ≤ 0.05 was considered statistically significant.

### Cytotoxicity assay

Intestinal porcine enterocytes cells (IPEC-J2) cells passages no 10–15 were used for viability test using standard MTT [3-(4,5-dimethylthiazol-2-yl)-2,5-diphenyltetrazolium bromide] assay^70^. Briefly, the IPEC-J2 cells were seeded at 12,000 cells/well in a 48-well plate for 24 h to reach 80–100% confluency. The cells were then washed with phosphate-buffered saline (PBS), and the test compounds were applied. Camptothecin was used as positive control in concentration 0–1.11 µg/mL. The negative control consisted of 0.35% DMSO, which corresponded to the concentration of DMSO used with the tested compounds. After 24 h of incubation with tested compounds, the cells were washed with PBS, and MTT solution in medium (0.5 mg/mL) was applied. After 1 h of incubation, the solution was discarded, whereas the residues dissolved in DMSO. The absorbance was measured at 570 nm (test) and 630 nm (reference). Each experiment was performed in three independent biological replicates. Statistical significance compared to untreated control samples was determined using the one-way analysis of variance test following multiple comparisons. All results were considered statistically significant if *p* < 0.05.

## Electronic supplementary material

Below is the link to the electronic supplementary material.

Supplementary Material 1

## Data Availability

The datasets used and analysed during the current study are available from the corresponding author on a reasonable request.
