# Supplementary material for: The assessment of chemical composition and biological activity of faba bean pods as a potential feed additive utilized in piglets nutrition
Source: Sci Rep. 2025 Jan 7;15:1234. doi: 10.1038/s41598-024-84925-9 (PMC11707233; doi:10.1038/s41598-024-84925-9)
Supplement: Supplementary file 1 — Supplementary Material 1 [file 41598_2024_84925_MOESM1_ESM.docx]

**SUPPLEMENTARY MATERIAL**

**Title:** The assessment of chemical composition and biological activity of faba bean pods as a potential feed additive utilized in piglets nutrition

**Authors:** Inna Vlasova^1^, Philip Krüsselmann^2^, Yuliia Kostenko^1^, Maciej Obrębski^1^, Sebastian Granica^1^, Wilfried Vahjen^2^, Jürgen Zentek^2^, Marcin Równicki^1*^, Jakub P. Piwowarski^1^

1 – Microbiota Lab, Department of Pharmaceutical Biology, Medical University of Warsaw, 1 Banacha St., 02-097, Warsaw, Poland

2 – Institute of Animal Nutrition, Department of Veterinary Medicine, Freie Universität Berlin, Berlin, Germany

*Correspondence to: [marcin.rownicki@wum.edu.pl](mailto:marcin.rownicki@wum.edu.pl)

**
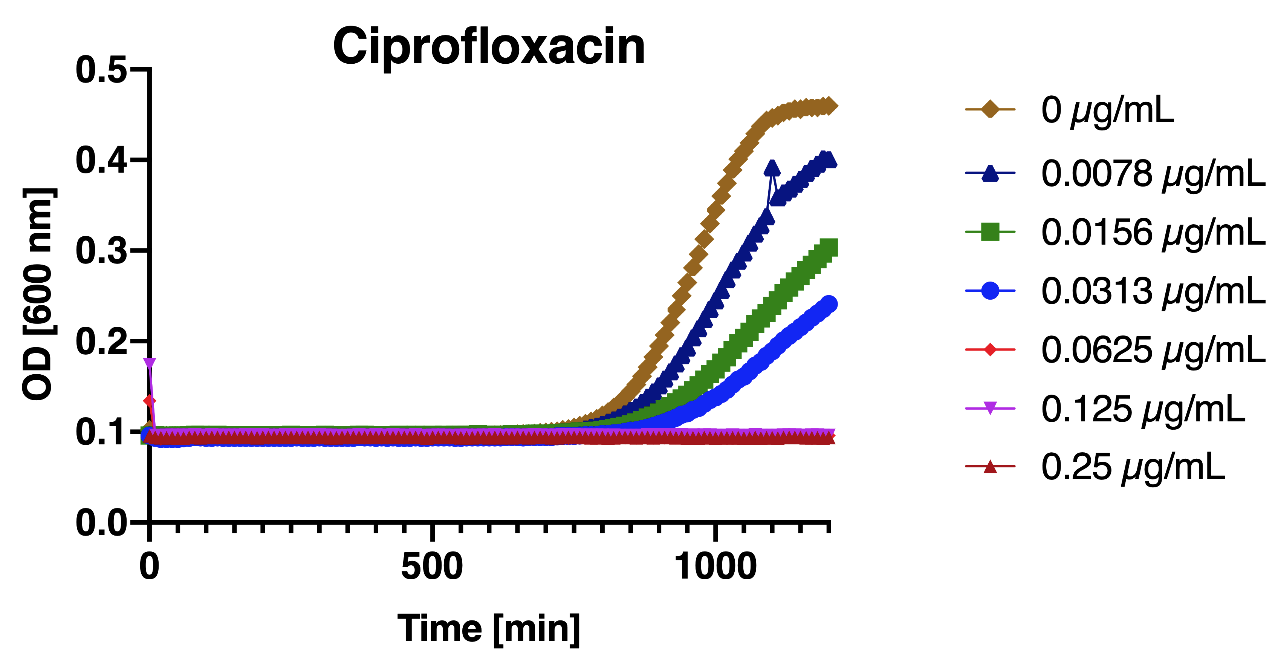
**

**Figure S1** Effect of different concentrations of ciprofloxacin on growth of *S. enterica*.

**
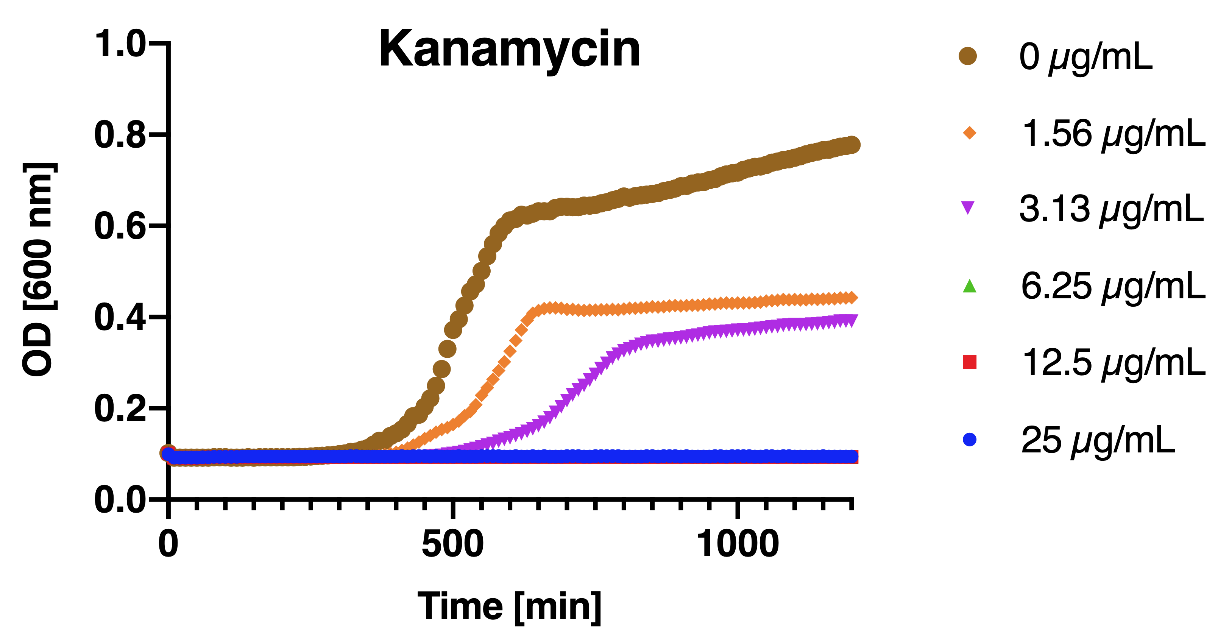
**

**Figure S2** Effect of different concentrations of kanamycin on growth of *E. coli*.

**
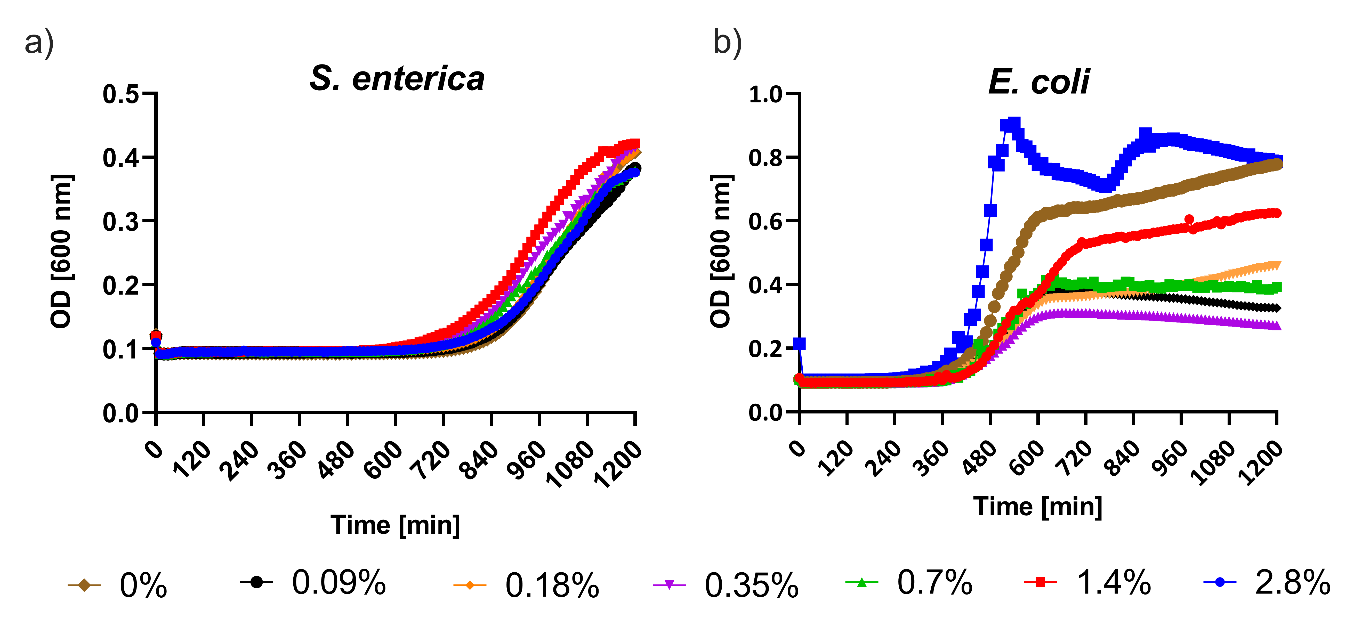
**

**Figure S3** Effect of different concentrations of methanol on growth of a) *S.* *enterica* and b) *E.* *coli.*
